# Body Mass Index Genetic Risk Score and Endometrial Cancer Risk

**DOI:** 10.1371/journal.pone.0143256

**Published:** 2015-11-25

**Authors:** Jennifer Prescott, Veronica W. Setiawan, Nicolas Wentzensen, Fredrick Schumacher, Herbert Yu, Ryan Delahanty, Leslie Bernstein, Stephen J. Chanock, Chu Chen, Linda S. Cook, Christine Friedenreich, Monserrat Garcia-Closas, Christopher A. Haiman, Loic Le Marchand, Xiaolin Liang, Jolanta Lissowska, Lingeng Lu, Anthony M. Magliocco, Sara H. Olson, Harvey A. Risch, Xiao-Ou Shu, Giske Ursin, Hannah P. Yang, Peter Kraft, Immaculata De Vivo

**Affiliations:** 1 Channing Division of Network Medicine, Department of Medicine, Brigham and Women’s Hospital and Harvard Medical School, Boston, Massachusetts, United States of America; 2 Program in Genetic Epidemiology and Statistical Genetics, Department of Epidemiology, Harvard T. H. Chan School of Public Health, Boston, Massachusetts, United States of America; 3 Department of Preventive Medicine, Keck School of Medicine, University of Southern California, Los Angeles, California, United States of America; 4 Division of Cancer Epidemiology and Genetics, National Cancer Institute, National Institutes of Health, Department of Health and Human Services, Bethesda, Maryland, United States of America; 5 Epidemiology Program, University of Hawaii Cancer Center, Honolulu, Hawaii, United States of America; 6 Department of Epidemiology and Public Health, Yale Cancer Center, Yale School of Public Health, New Haven, Connecticut, United States of America; 7 Division of Epidemiology, Department of Medicine and Vanderbilt-Ingram Cancer Center, Vanderbilt University School of Medicine, Nashville, Tennessee, United States of America; 8 Beckman Research Institute, City of Hope, Duarte, California, United States of America; 9 Division of Public Health Sciences, Fred Hutchinson Cancer Research Center and Department of Epidemiology, School of Public Health, University of Washington, Seattle, Washington, United States of America; 10 University of New Mexico, Albuquerque, New Mexico, United States of America; 11 Division of Cancer Care, Department of Population Health Research, Alberta Health Services–Cancer Control Alberta, Calgary, Alberta, Canada; 12 Division of Genetics and Epidemiology, Institute of Cancer Research, Sutton, United Kingdom; 13 Memorial Sloan Kettering Cancer Center, New York, New York, United States of America; 14 Department of Epidemiology and Cancer Prevention, Cancer Center and M Sklodowska-Curie Institute of Oncology, Warsaw, Poland; 15 H. Lee Moffitt Cancer Centre, Tampa, Florida, United States of America; 16 Cancer Registry of Norway, Oslo, Norway; 17 Department of Nutrition, Institute of Basic Medical Sciences, University of Oslo, Oslo, Norway; National Health Research Institutes, TAIWAN

## Abstract

Genome-wide association studies (GWAS) have identified common variants that predispose individuals to a higher body mass index (BMI), an independent risk factor for endometrial cancer. Composite genotype risk scores (GRS) based on the joint effect of published BMI risk loci were used to explore whether endometrial cancer shares a genetic background with obesity. Genotype and risk factor data were available on 3,376 endometrial cancer case and 3,867 control participants of European ancestry from the Epidemiology of Endometrial Cancer Consortium GWAS. A BMI GRS was calculated by summing the number of BMI risk alleles at 97 independent loci. For exploratory analyses, additional GRSs were based on subsets of risk loci within putative etiologic BMI pathways. The BMI GRS was statistically significantly associated with endometrial cancer risk (*P* = 0.002). For every 10 BMI risk alleles a woman had a 13% increased endometrial cancer risk (95% CI: 4%, 22%). However, after adjusting for BMI, the BMI GRS was no longer associated with risk (per 10 BMI risk alleles OR = 0.99, 95% CI: 0.91, 1.07; *P* = 0.78). Heterogeneity by BMI did not reach statistical significance (*P* = 0.06), and no effect modification was noted by age, GWAS Stage, study design or between studies (*P*≥0.58). In exploratory analyses, the GRS defined by variants at loci containing monogenic obesity syndrome genes was associated with reduced endometrial cancer risk independent of BMI (per BMI risk allele OR = 0.92, 95% CI: 0.88, 0.96; *P* = 2.1 x 10^−5^). Possessing a large number of BMI risk alleles does not increase endometrial cancer risk above that conferred by excess body weight among women of European descent. Thus, the GRS based on all current established BMI loci does not provide added value independent of BMI. Future studies are required to validate the unexpected observed relation between monogenic obesity syndrome genetic variants and endometrial cancer risk.

## Introduction

Endometrial cancer incidence is projected to surpass colorectal cancer to become the 3^rd^ leading cancer site among U.S. women by 2030 [1]. Excess adiposity, a well-established risk factor for endometrial cancer [2], is mainly considered a consequence of modifiable lifestyle choices. Genome-wide association studies (GWAS) have identified common variants that predispose individuals to a higher body mass index (BMI) [3]. Single nucleotide polymorphisms (SNPs) at a few BMI loci have been examined in relation to endometrial cancer risk [4–9]. In a prior study by the Epidemiology of Endometrial Cancer Consortium (E2C2), the rs9939609 A allele at the fat mass and obesity-associated (*FTO*) locus was associated with endometrial cancer risk, a relation mediated by BMI. In contrast, the obesity risk variant, rs17782313, at the melanocortin 4 receptor (*MC4R*) locus was not associated with risk regardless of whether the model was adjusted for BMI [7]. In another study, a variant at the *FTO* (rs12927155) locus that was most statistically significantly associated with endometrial cancer risk in the Polish Endometrial Case-Control Study (PECS) was not associated with risk in replication studies [6]. Thus far, only one GWAS endometrial cancer risk locus has been identified [HNF1 homeobox B (*HNF1B*)] [8, 10, 11] that is independent of BMI and has been replicated in independent populations of European and non-European descent [8, 9, 11].

Common genetic variants generally account for a very small proportion of variation in associated phenotypes. Thus, extremely large sample sizes may be required to observe statistically significant associations for individual SNPs [12]. Alternatively, a composite genotype score based on the joint effect of risk loci may contribute substantially to disease risk [13] and may be used to examine shared genetic background between obesity and endometrial cancer risk. One study found that summing the number of adiposity-increasing alleles from 26 unique loci into a genetic risk score (GRS) was associated with increased endometrial cancer risk among Chinese women. The obesity GRS remained statistically significantly associated with endometrial cancer risk even after adjusting for BMI at study enrollment [14]. A more recent publication observed increased endometrial cancer risk associated with a composite BMI-increasing GRS based on 32 unique loci among individuals of European ancestry, but did not assess whether the association was independent of BMI [15]. Thus, our goal was to explore whether a BMI GRS is associated with endometrial cancer risk independent of BMI among women of European ancestry.

## Materials and Methods

### Study Population

Participants in this study included women of European descent from the discovery (Stage I) and replication (Stage II) populations of a GWAS of Type I endometrial cancer conducted by the E2C2 [11]. Type I tumors are comprised of endometrioid (ICDO codes 8380, 8381, 8382, and 8383), adenocarcinoma tubular (codes 8210 and 8211), papillary adenocarcinoma (codes 8260, 8262, and 8263), adenocarcinoma with squamous metaplasia (code 8570), mucinous adenocarcinoma (codes 8480 and 8481), and adenocarcinoma not otherwise specified (code 8140). The current analysis includes five population-based case-control studies [Alberta Health Services (AHS); Connecticut Endometrial Cancer Study (CECS); Estrogen, Diet, Genetics, and Endometrial Cancer (EDGE); Fred Hutchinson Cancer Research Center case-control studies (FHCRC); PECS] and four case-control studies nested within prospective cohorts [Multiethnic Cohort (MEC); California Teachers Study (CTS); Nurses’ Health Study (NHS); Prostate, Lung, Colorectal, and Ovarian Cancer Screening Trial (PLCO)] for a total of 3,376 primary incident invasive endometrial cancer cases and 3,867 controls who were free of endometrial cancer and did not have histories of hysterectomy. Study design characteristics for each contributing study are summarized in S1 Table, with details previously published elsewhere [9, 16–26].

### Ethics Statement

Each study was approved by the host institutions’ Institutional Review Boards [CECS: Yale University, Connecticut Department of Public Health Human Investigation Committee, the 28 participating Connecticut hospitals; CTS: Cancer Prevention Institute of California, City of Hope National Medical Center, University of Southern California, University of California, Irvine, California Health and Human Services Agency; FHCRC: Fred Hutchinson Cancer Research Center; MEC: University of Southern California, University of Hawaii; NHS: Brigham and Women’s Hospital; PECS: U.S. National Cancer Institute, M. Sklodowska Curie Institute of Oncology and Cancer Center in Warsaw, Institute of Occupational Medicine in Lodz; PLCO: National Cancer Institute, the 10 participating screening centers; AHS: University of Calgary, Alberta Cancer Board; EDGE: New Jersey Department of Health and Senior Services, Memorial Sloan Kettering Cancer Center and University of Medicine, Dentistry of New Jersey (UMDNJ) Robert Wood Johnson Medical School] and appropriate permission for the pooled analysis was obtained. Written informed consent was obtained from all participants according to each study’s approved protocol except for women participating in MEC and NHS. Return of the MEC and NHS mailed self-administered questionnaires was voluntary. Thus, receipt of a completed questionnaire was considered as evidence of a desire to participate in the study and was taken as a formal indication of consent by the respective Institutional Review Boards.

### Genotyping

Cases and matched controls from CECS, FHCRC, MEC, CTS, and NHS studies were genotyped on the HumanOmniExpress Beadchip (~700K markers; Illumina Inc., San Diego, CA), and AHS and EDGE samples were genotyped on the HumanExome Beadchip (~250K markers; Illumina Inc.) at the University of Southern California. Biospecimens from PLCO cases were genotyped on the OmniExpress chip, PLCO controls were genotyped on the HumanOmni2.5 Beadchip (~2.5 million markers; Illumina Inc.), and PECS cases and controls were previously genotyped on Human660W-Quad Beadchip (~660K markers; Illumina Inc.) at the NCI Cancer Genomics Research Laboratory. Sample and genotyping quality control metrics are described in De Vivo et al. [11]. Briefly, GWAS Stage I participants with <80% European genetic ancestry and Stage II participants who self-reported as non-white were excluded. In both Stage I and II, SNPs with completion rates <90%, minor allele frequencies <1% in each study, or out of Hardy-Weinberg equilibrium (*P* < 1 x 10^−4^ in Stage I, *P* < 1 x 10^−5^ in Stage II) among controls were removed. After these quality control filters were applied, >524K genotyped SNPs remained in each Stage I study. Approximately 31 million SNPs were imputed separately by platform using MACH (v.1.0.18.c) software [27, 28] (r^2^ > 0.80) and the 1000 Genomes Project March 2012 release as the reference panel. Within each platform, SNPs with low imputation quality (r^2^ < 0.90) were replaced by selecting values from participants genotyped on the other platform(s) using random hot deck imputation and case status as the matching factor.

### Genetic Risk Scores (GRS)

Risk variants were chosen specifically based on their established associations with BMI. SNPs selected for the BMI GRS were 97 independent loci validated and/or identified at the genome-wide significance level (*P* < 5 x 10^−8^) from a genome-wide meta-analysis of BMI that included 339,224 individuals [3].

Counts or imputed dosage of each BMI-increasing risk allele (range: 0 to 2) were exported from the imputed data sets. For the main analysis, we generated a GRS assuming each BMI-associated SNP contributes equally to increased endometrial cancer risk. The GRS is calculated by summing the number of risk alleles across loci, producing a score with a potential maximum of 194 for the total number of BMI-increasing risk alleles. As a sensitivity analysis, we additionally generated a weighted GRS by incorporating the added step of multiplying each SNP by the relative effect sizes (β-coefficient) reported by Locke et al. [3] before summing the products to account for the strength of prior associations. Detailed calculations for the weighted genetic scores have been described previously [13, 29]. For exploratory analyses, we additionally created BMI-increasing GRSs based on SNPs in the biologic pathways underlying BMI etiology identified by the Genetic Investigation of Anthropometric Traits (GIANT) Consortium [3]: central nervous system processes [rs11583200 (*ELAVL4*), rs7899106 (*GRID1*), rs13078960 (*CADM2*), rs7141420 (*NRXN3*), rs3101336 (*NEGR1*), rs3736485 (*SCG3; DMXL2*)], monogenic obesity syndromes [rs6567160 (*MC4R*), rs11030104 (*BDNF*), rs7164727 (*BBS4; LOC100287559*), rs10182181 (*POMC; ADCY3*)], extreme/early obesity [rs3888190 (*SH2B1; ATP2A1*), rs3101336 (*NEGR1*)], lipid biology and/or adipogenesis [rs7903146 (*TCF7L2*), rs2287019 (*GIPR; QPCTL*), rs2176040 (*IRS1; LOC646736*), rs9400239 (*FOXO3*), rs6465468 (*ASB4*), rs12940622 (*RPTOR*), rs1808579 (*NPC1; C18orf8*), rs17203016 (*CREB1*), rs4787491 (*FAM57B; INO80E*), rs2650492 (*APOBR; SBK1*), rs2176598 (*HSD17B12*)], RNA binding/processing proteins [rs11165643 (*PTBP2*), rs11583200 (*ELAVL4*), rs3817334 (*CELF1; MTCH2*), rs2033732 (*RALYL*)], MAP kinase signaling [rs16951275 (*MAP2K5*), rs4787491 (*MAPK3; INO80E*)], and cell proliferation/survival [rs7138803 (*FAIM2; BCDIN3D*), rs13191362 (*PARK2*), rs12429545 (*OLFM4*)].

### Statistical Analysis

Information on risk factors for endometrial cancer, collected by each study using structured questionnaires, was obtained from the E2C2 coordinating center, which previously compiled and harmonized the data. Six individuals missing age at diagnosis (5 cases) or reference age (1 control) were excluded from the present analysis. We used pooled unconditional logistic regression to estimate per risk allele odds ratios (OR) and 95% confidence intervals (CI) associated with endometrial cancer risk for each BMI variant (S2 Table) and GRS. Linear regression was used to model the association of the BMI GRS with BMI. All analyses were adjusted for age at diagnosis/reference and study. BMI (continuous in kg/m^2^ with missing indicator for N = 86) was also considered as a potential intermediate. Wald statistics were used to estimate trend *P*-values. A binomial statistic assessed whether the number of BMI-increasing risk alleles associated with elevated endometrial cancer risk was more than expected by chance. BMI GRS categories were defined using the interquartile range among control participants. Likelihood ratio statistics assessed heterogeneity in risk associated with the BMI GRS by median age at diagnosis (<62, ≥62 years), BMI (<25, 25 to <30, ≥30 kg/m^2^), GWAS Stage (I, II), and study design (case-control, cohort). The Q test was used to assess heterogeneity between studies. All *P*-values were two-sided; *P*-values <0.05 were considered statistically significant. We used SAS Version 9.3 software (SAS Institute, Cary, NC) for all analyses except the binomial statistic, which used R Version 3.1.1 (R Foundation for Statistical Computing, Vienna, Austria).

## Results

Our analytic population consisted of 3,376 endometrial cancer case and 3,867 control participants of European ancestry from Stages I (N = 5,471) and II (N = 1,772) of the E2C2 endometrial cancer GWAS. Selected characteristics of women by GWAS Stage population and case-control status are presented in Table 1.

**Table 1:** Selected Population Characteristics by Genome-Wide Association Study Stage and Case-Control Status Among Women of European Ancestry.

	GWAS Stage I		GWAS Stage II	
	Cases	Controls	Cases	Controls
Total N	2,695	2,776	681	1,091
Mean age (SD)	62.3 (8.3)	60.8 (9.7)	59.7 (9.3)	59.9 (10.5)
Mean BMI (SD)	29.7 (7.6)	26.1 (5.3)	32.4 (8.4)	27.8 (5.6)
Diabetes (%)	11.7	4.7	12.3	6.9
Ever used hormone therapy (%)	48.2	46.4	37.6	42.5
Mean BMI GRS (SD)	91.8 (6.2)	91.4 (6.2)	91.9 (6.1)	91.4 (6.2)

Abbreviations: BMI, body mass index; GRS, genetic risk score; GWAS, genome-wide association study; N, sample size; SD standard deviation.

Among control participants, the BMI GRS was statistically significantly associated with increasing BMI (β = 0.14 per unit of kg/m^2^, SE = 0.01; *P* = 1.6 x 10^−24^). After adjustment for age and study, out of 97 BMI risk variants, 60 displayed estimates in directions consistent with increased risk of endometrial cancer (binomial *P* = 0.007; S2 Table). Likewise, the BMI GRS was positively associated with endometrial cancer risk in a multivariable logistic regression model adjusted for age and study (Table 2). For each additional 10 BMI-increasing risk alleles, risk of developing endometrial cancer increased by 13% (95% CI: 4%, 22%; *P* = 0.002). However, after additionally adjusting for BMI, the BMI GRS was no longer associated with endometrial cancer risk (*P* = 0.78). We observed a similar pattern when restricting analyses to cases with endometrioid histology (N = 2,094).

**Table 2:** Body Mass Index Genetic Risk Score and Endometrial Cancer Risk Among Women of European Ancestry.

	Model 1^a^			Model 2^b^		
	OR^c^	95% CI	*P* trend	OR^c^	95% CI	*P* trend
*All Type I Endometrial Tumors (Case N = 3*,*376)*	1.13	1.04, 1.22	0.002	0.99	0.91, 1.07	0.78
*Endometrioid Tumors (Case N = 2*,*094)*	1.11	1.02, 1.21	0.02	0.95	0.86, 1.04	0.28
*All Type I Endometrial Tumors among never hormone users (Case N = 1*,*820)*	1.15	1.04, 1.28	0.007	0.97	0.86, 1.08	0.55

Abbreviations: BMI, body mass index; CI, confidence interval; OR, odds ratio.

^a^ Unconditional logistic regression models adjusted for age at diagnosis and study were used to estimate odds ratios and 95% confidence intervals

^b^ Unconditional logistic regression models were additionally adjusted for BMI

^c^ Per 10 BMI risk alleles

**Fig 1:**
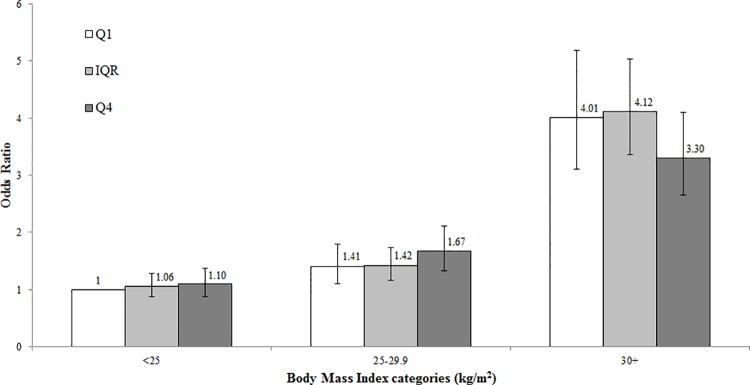
Endometrial Cancer Risk within BMI and BMI GRS Subgroups. Data represent odds ratios and 95% confidence intervals of endometrial cancer for quartile 1 (Q1; 67.5–87.1 risk alleles), the interquartile range (IQR; 87.2–95.5 risk alleles), and quartile 4 (Q4; 95.6–115.3 risk alleles) categories of the BMI GRS among normal weight (<25 kg/m^2^), overweight (25–29.9 kg/m^2^), and obese (30+ kg/m^2^) women.

As excess adipose tissue and menopausal estrogen therapy have previously been found to modify genetic and lifestyle risk factor associations with endometrial cancer [30–34], presumably by increasing exposure to circulating estrogens, we explored whether the BMI GRS was more predictive of endometrial cancer risk within subgroups of the population (Fig 1). The BMI GRS was not associated with endometrial cancer risk among normal weight women (<25 kg/m^2^; *P-trend* = 0.40), positively associated among overweight women (25–29.9 kg/m^2^; *P-trend* = 0.06), and inversely associated with risk among obese women (30+ kg/m^2^; *P-trend* = 0.01). However, the test for heterogeneity in effect estimates did not reach statistical significance (*P-heterogeneity* = 0.06). We did not find evidence for heterogeneity by age at diagnosis, GWAS Stage, study design, or between studies (*P-heterogeneity* ≥ 0.58; data not shown). When the analysis was restricted to women who never used menopausal hormones (N = 1,820 cases; N = 2,115 controls), the BMI GRS was associated with endometrial cancer risk in models adjusted for age and study, but not after additionally adjusting for BMI (Table 2). Results remained the same when analyses were repeated using the weighted BMI GRS (S3 Table).

We explored whether SNP variations in the diverse pathways identified by the GIANT Consortium [3] were associated with endometrial cancer risk by creating GRSs based on subsets of BMI-increasing risk variants (Table 3). The GRS based on loci near RNA binding/processing protein genes increased risk of endometrial cancer prior to adjusting for BMI, but the association was attenuated after including BMI in the model. In contrast, a GRS of loci containing monogenic obesity syndrome (MOS) genes was inversely associated with risk (per risk allele OR = 0.94; 95% CI: 0.91, 0.98) that strengthened after controlling for BMI (OR = 0.92; 95% CI: 0.88, 0.96). Results were similar when analyses were restricted to cases with endometrioid histology. The reduced risk associated with the MOS GRS was observed among overweight (OR = 0.88; 95% CI: 0.82, 0.95) and obese (OR = 0.90; 95% CI: 0.84, 0.97) individuals, but not among normal weight women (OR = 0.97; 95% CI: 0.91, 1.04; *P-heterogeneity* = 0.03). The association did not differ between studies (*P-heterogeneity* = 0.17). Each of the 4 BMI-increasing risk alleles of the MOS GRS was inversely associated with endometrial cancer risk (S2 Table). The MOS GRS was positively associated with BMI among controls (β = 0.31 per unit of kg/m^2^, SE = 0.07; *P* = 6.7 x 10^−6^).

**Table 3:** Genetic Risk Scores Based on Biologic Single Nucleotide Polymorphism Subsets and Endometrial Cancer Risk Among Women of European Ancestry.

	Model 1^a^			Model 2^b^		
Genetic risk scores	OR	95% CI	*P* trend	OR	95% CI	*P* trend
Central Nervous System	0.99	0.96, 1.02	0.56	0.98	0.95, 1.02	0.32
Monogenic Obesity Syndromes	0.94	0.91, 0.98	0.002	0.92	0.88, 0.96	2.1 x 10^−5^
Extreme/Early Obesity	1.04	0.99, 1.10	0.08	1.02	0.97, 1.07	0.45
Lipid Biology and/or Adipogenesis	1.00	0.98, 1.02	0.92	0.99	0.97, 1.01	0.38
RNA Binding/Processing Proteins	1.04	1.00, 1.07	0.04	1.02	0.99, 1.06	0.19
MAP Kinase Signaling Pathway	1.01	0.97, 1.07	0.60	1.01	0.96, 1.07	0.69
Cell Proliferation/Survival	1.04	0.99, 1.09	0.13	1.03	0.98, 1.08	0.31

Abbreviations: BMI, body mass index; CI, confidence interval; OR, odds ratio.

^a^ Unconditional logistic regression models adjusted for age at diagnosis and study were used to estimate per risk allele odds ratios and 95% confidence intervals

^b^ Unconditional logistic regression models were additionally adjusted for BMI

## Discussion

We conducted a study among women of European ancestry to look for a shared genetic background between obesity and endometrial cancer using a composite BMI genotype score. Risk for developing endometrial cancer increased by 13% for each interval of 10 BMI-increasing alleles possessed. However, the BMI GRS associations with endometrial cancer in our study were completely attenuated after adjusting for BMI at diagnosis. In exploratory analyses of etiologic pathways underlying obesity, we observed an unexpected BMI-independent reduced risk of endometrial cancer associated with BMI-increasing risk alleles located near MOS genes. Overall, our results suggest that a GRS based on current established BMI loci does not provide added value independent of BMI in predicting Type I endometrial cancer risk among women of European ancestry.

A recent analysis among individuals of European ancestry observed highly statistically significant positive associations between a 32-locus BMI GRS and BMI (*P* = 3.3 x 10^−22^) and endometrioid endometrial cancer risk (*P* = 1.2 x 10^−6^), relationships that are consistent in our population. However, the authors did not report whether the association between the BMI GRS and endometrioid endometrial cancer risk was independent of BMI [15]. The study conducted in a Chinese population by Delahanty et al. observed that a 26-locus obesity GRS was associated with endometrial cancer risk independent of BMI. Different criteria were used in the selection of risk variants [14], such that only 14 loci were common between the Delahanty study and ours; this could have led to discrepant results in the overall BMI GRS associations. SNPs selected for the present analysis were variants associated with BMI at the *P* < 5 x 10^−8^ significance level as reported by the most recent GIANT Consortium GWAS meta-analysis [3]. Delahanty et al. searched for variants associated with BMI, obesity, waist-to-hip ratio, and/or adiposity at *P* < 5 x 10^−7^ within the National Human Genome Resource Institute GWAS catalog [14]. This may partly explain the weaker relation between their obesity GRS and BMI (β = 0.07 per kg/m^2^) [14] compared to the association in our study (β = 0.14 per kg/m^2^). However, by including adiposity-related loci that are independent of BMI, Delahanty et al. may have produced a more informative GRS for disease risk.

In an exploratory analysis, we observed an unexpected independent inverse association between endometrial cancer and a GRS based on BMI-increasing common risk alleles at loci containing MOS genes. We did not observe between-study heterogeneity; each risk variant of the MOS GRS showed an inverse association with risk, and we confirmed the positive association between the MOS GRS and BMI. MOS genes play a physiologic role in neurodevelopment and regulation of the hypothalamic leptin-melanocortin system [35]. To our knowledge, the influence of this pathway on cancer development independent of energy balance has not been established, suggesting our results may be due to chance and require validation.

Strengths of our study included the large number of genotyped subjects from well-designed endometrial cancer studies and the genome-wide significance cut-off of *P* < 5 x 10^−8^ used to select BMI loci in an effort to minimize inclusion of false positives that could dilute genetic effects. However, use of the stringent threshold very likely excluded other true variants associated with BMI. Genetic loci selected for this analysis explain only 2.7% of the estimated 21% inter-individual variation in BMI accounted for by common genetic variants [3]. By restricting the analysis to women of European ancestry, we reduced confounding by population stratification, but also limited the generalizability of our study results. Thus, replication in women of non-European ancestry is warranted.

In summary, based on the current list of established genetic loci, BMI risk alleles as a whole do not increase endometrial cancer risk independent of BMI among women of European ancestry. The observation that common BMI-increasing genetic variants near MOS genes may reduce endometrial cancer risk requires validation. Progress in identifying biological roles for new and existing BMI loci could provide much needed insight into this disease.

## Supporting Information

S1 Table Characteristics of 9 Studies Included in the Analysis. Details of study populations included in the current analysis.
(XLSX)

S2 Table Body Mass Index-Increasing Risk Allele Associations with Endometrial Cancer Risk Among Women of European Ancestry. Odds ratios and 95% confidence intervals of individual SNP associations with endometrial cancer risk in models with and without adjustment for BMI.
(XLSX)

S3 Table Weighted Body Mass Index Genetic Risk Score and Endometrial Cancer Risk Among Women of European Ancestry. Odds ratios and 95% confidence intervals of the weighted BMI GRS associated with endometrial cancer risk in models with and without adjustment for BMI.
(DOCX)
