# Supplementary material for: Body Mass Index Genetic Risk Score and Endometrial Cancer Risk
Source: PLoS One. 2015 Nov 25;10(11):e0143256. doi: 10.1371/journal.pone.0143256 (PMC4659592; doi:10.1371/journal.pone.0143256)
Supplement: S3 Table — Odds ratios and 95% confidence intervals of the weighted BMI GRS associated with endometrial cancer risk in models with and without adjustment for BMI. (DOCX) [file pone.0143256.s003.docx]

**S3 Table. Weighted Body Mass Index Genetic Risk Score and Endometrial Cancer Risk Among Women of European Ancestry.**

|  | Model 1^a^ | | |  | Model 2^b^ | | |
| --- | --- | --- | --- | --- | --- | --- | --- |
|  | OR^c^ | 95% CI | *P* trend |  | OR^c^ | 95% CI | *P* trend |
| *All Type I Endometrial Tumors (Case N=3,376)* | 1.16 | 1.08, 1.25 | 0.0001 |  | 1.00 | 0.92, 1.08 | 0.96 |
| *Endometrioid Tumors (Case N=2,094)* | 1.16 | 1.06, 1.27 | 0.0008 |  | 0.97 | 0.89, 1.07 | 0.56 |
| *All Type I Endometrial Tumors among never hormone users (Case N=1,820)* | 1.22 | 1.10, 1.35 | 0.0002 |  | 0.99 | 0.89, 1.11 | 0.86 |

Abbreviations: BMI, body mass index; CI, confidence interval; OR, odds ratio.

^a^ Unconditional logistic regression models adjusted for age at diagnosis and study were used to estimate odds ratios and 95% confidence intervals

^b^ Unconditional logistic regression models were additionally adjusted for BMI

^c^ Per 10 BMI risk alleles
